# Invasive freshwater snails are less sensitive to population density than native conspecifics

**DOI:** 10.1002/ece3.11161

**Published:** 2024-05-20

**Authors:** Briante Shevon Lewis Najev, Maurine Neiman

**Affiliations:** ^1^ Department of Biology University of Iowa Iowa City Iowa USA; ^2^ Department of Gender, Women's and Sexuality Studies University of Iowa Iowa City Iowa USA

**Keywords:** density dependence, invertebrates, non‐native species, population density, population dynamics, *Potamopyrgus antipodarum*

## Abstract

Understanding how and why some species or lineages become invasive is critically important for effectively predicting and mitigating biological invasions. Here, we address an important unanswered question in invasion biology: do key life‐history traits of invasive versus native lineages *within a species* differ in response to key environmental stressors? We focus on the environmental factor of population density, which is a fundamental characteristic of all populations, and investigate how changes in density affect native versus invasive *Potamopyrgus antipodarum* (New Zealand mudsnail). *P. antipodarum* has invaded 39 countries and detrimentally affects invaded environments. Previous studies of native and invasive populations and from laboratory experiments have demonstrated that growth and reproduction of *P*. *antipodarum* is sensitive to population density, though whether and how this sensitivity varies across native versus invasive lineages remains uncharacterized. We quantified individual growth rate and reproduction in *P*. *antipodarum* from multiple distinct native and invasive lineages across three different population density treatments. The growth of native but not invasive lineages decreased as density increased. There was no differential effect of density treatment on embryo *production* of invasive versus native snails, but a significantly higher proportion of snails were reproductive in high density compared to intermediate density for invasive lineages. In native lineages, there were no significant differences in the relative frequency of reproductive snails across density treatments. While the extent to which these results from our laboratory study can be extrapolated to the more complex natural world remain unclear, our findings are consistent with a scenario where differential sensitivity to population density could help explain why some lineages become successful invaders. Our findings also align with previous studies that show that invasive *P*. *antipodarum* lineages exhibit a relatively wide range of tolerance to environmental stressors.

## Graphical abstract

Species invasion can negatively affect natural ecosystems, so understanding how and why lineages become invasive is critically important to effectively predict potential future invasions. We address whether key life‐history traits across invasive versus native lineages of a New Zealand freshwater snail species differ in response to population density. We found that life‐history trait values in invasive lineages were less variable across population density treatments than for native counterparts, consistent with a scenario where differential sensitivity to population density could help explain why some lineages become successful invaders.

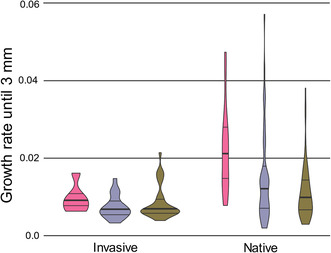

## 1 INTRODUCTION

All biological invasions start with introduction, which is defined as entry of a foreign species into a novel ecosystem via human action (Jaksic & Castro, 2021; Richardson et al., 2000). Recently introduced populations often have low population density (Geburzi & McCarthy, 2018), which can translate into relatively high risk that a population can be extinguished by phenomena including but not limited to anthropogenic influences (e.g., Forseth et al., 2017; Frick et al., 2020; Matthies et al., 2004), disease (e.g., Trathan et al., 2015), and natural disasters (e.g., Ameca y Juárez et al., 2015). Because failed introduction does not result in successful biological invasion, multiple introduction events are often required for establishment (Tabak et al., 2018). If introduction is successful, the resulting established invasive populations are faced with biological challenges that differ from those experienced during introduction (Sakai et al., 2001), such as high intraspecific population density (e.g., Paton et al., 2019; Stadler et al., 2023). Together, these phenomena suggest that understanding how members of invasive populations respond to population density can provide important insights into biological invasion.

We focus on intraspecific population dynamics of biological invasions because many invasions involve more than one conspecific individual (reviewed in Cassey et al., 2018), and the population size of invasive species can ebb and flow (reviewed in Simberloff & Gibbons, 2004). Interactions between conspecifics can profoundly influence morphology (e.g., Bhavanam & Trewick, 2022), reproduction (e.g., Kobayashi, 2019), competition for mates (e.g., Ode et al., 2022), and other key aspects of population dynamics of potentially invasive species (e.g., Karatayev et al., 2015). In particular, population density might reasonably affect intraspecific competition, which can in turn influence dispersal (e.g., Grabowska et al., 2019), behavior (e.g., Adey & Larson, 2021), and life‐history traits (e.g., McCann et al., 2019; Pervez & Sharma, 2021; Ward et al., 2013). Here, we evaluate whether key life‐history traits (growth rate, embryo production, embryo presence vs. absence) are differentially affected by conspecific population density in invasive versus native *P*. *antipodarum* by manipulating the population density experienced by juvenile representatives of multiple native and invasive snail lineages and quantifying their individual growth rate and embryo production. Better performance of invasive lineages under either low‐ or high‐density conditions, manifesting as a higher growth rate, more embryos produced, or increased likelihood of being reproductively active, relative to native counterparts, is consistent with a scenario where density‐dependent mechanisms might contribute to invasion success.

### 1.1 Does life‐history trait variation help drive invasion in a destructive freshwater snail?

In this paper, we evaluate whether and how the life‐history traits of asexual lineages of *Potamopyrgus antipodarum* (Gastropoda: Tateidae; J.E. Gray, 1843), an invasive freshwater snail, are regulated by population density. *Potamopyrgus antipodarum*, colloquially known as the New Zealand mudsnail, is a globally invasive freshwater snail found in fresh and brackish waters in their native New Zealand range. These snails are a powerful model for biological invasion studies because they have recently invaded all continents except Antarctica (Alonso & Castro‐Díez, 2012; Collado, 2014; Taybi et al., 2021). In these novel environments, *P*. *antipodarum* can drastically change the community dynamics of invaded ecosystems by competing with native biota (Kerans et al., 2005), replacing the native primary consumers (Rakauskas et al., 2018), and consuming most primary production (Hall Jr. et al., 2003). Populations of *P*. *antipodarum* in their native range are characterized by the coexistence of sexual and asexual individuals (Fox et al., 1996; Neiman et al., 2011), but invasive populations of *P*. *antipodarum* are exclusively asexual (Tatara et al., 2014; Wallace, 1992). Only a handful of asexual lineages of *P*. *antipodarum* have invaded worldwide (Donne et al., 2020), though most of these lineages ultimately originated from the North Island of New Zealand (Donne et al., 2020; Städler et al., 2005), which features the highest genetic diversity for New Zealand *P*. *antipodarum* (Neiman & Lively, 2004). The global success of these few invasive lineages is especially notable because of the lack of access to novel genetic diversity expected for asexual populations, raising the question of how these genetically depauperate founding populations can be so successful.

Part of the answer to this question could lie in the fact that *P*. *antipodarum* is characterized by traits that could facilitate biological invasions, including the aforementioned asexual reproduction (Hershler et al., 2010) as well as marked phenotypic plasticity (Kistner & Dybdahl, 2013; Levri et al., 2014; Verhaegen et al., 2018; Xu et al., 2022), tolerance to a wide range of environmental conditions (Alonso & Camargo, 2009; Cieplok et al., 2023; Leclair & Cheng, 2011; Neiman & Krist, 2016; Romero‐Blanco & Alonso, 2019), and resistance to predation (Levri et al., 2017; Rakauskas et al., 2016). New Zealand populations of *P*. *antipodarum* harbor wide heritable variation in critical life‐history traits (Larkin et al., 2016) and are also very sensitive to population density (Neiman, 2006; Neiman et al., 2013; Zachar & Neiman, 2013), chemical interference (Levy et al., 1973), resource competition (Cope & Winterbourn, 2004), and predation risks (Steele & Forrester, 2005). These properties of *P*. *antipodarum* raise the intriguing possibility that at least some of the success of this asexual invader might be linked to differential life‐history trait responses to population density in native versus invasive lineages.

With specific regard to our focus here, multiple introduced *P*. *antipodarum* populations have experienced sudden and drastic fluxes in population size (Greenwood et al., 2020) as well as more subtle fluctuations over longer periods of time (Cimino et al., 2020; Dorgelo, 1987; Strayer et al., 2017). Some introduced *P*. *antipodarum* populations have ultimately failed to establish despite rapidly reaching high densities early in the invasion process (Moore et al., 2012). Indeed, introduced populations of *P*. *antipodarum* can attain remarkably high density, ranging from 19,000/m^2^ (Nelson & Armstrong, 2022), 299,000/m^2^ (Kerans et al., 2005), and to 500,000/m^2^ (Hall Jr. et al., 2003), hinting that these population crashes could be related to density‐dependent mechanisms (Nelson & Armstrong, 2022). Here, we extend this logic by hypothesizing that successfully invading asexual lineages might be relatively insensitive to population density compared to native lineages. By this reasoning, non‐successful colonist *P*. *antipodarum* lineages might also be expected to exhibit similar responses to population density as native lineages.

## 2 METHODS

### 2.1 Snail sampling and culture establishment/maintenance

Native‐range snails (hereafter, “native” lineages/snails) were collected with kick nets from the shallow regions of three New Zealand freshwater lakes: South Mavora, Okareka, and Grasmere. We collected invasive snails (hereafter, “invasive” lineage/snails) from three North American freshwater stream sites in Pennsylvania, Wyoming, and New York. New York snails were collected in a Syracuse stream by Ed Levri in 2019. More background information on the Pennsylvania and Wyoming snails can be found by referring to the Spring Creek (“PA”) and Polecat Creek (“Pc”) collections, respectively, in Donne et al. (2020). In short, Donne et al. (2020) show that while the Spring Creek and Polecat Creek snails harbor distinct nuclear genotypes, both belong to the “US1” lineage. To maintain consistency with previous work that has referred to the Polecat Creek‐collected snails as “US1” (Levri et al., 2014), we continue that tradition here. All field‐collected snails were then transferred to the University of Iowa, where individual females were isolated and used to found laboratory cultures. The ploidy, coupled with reproductive mode, of the New Zealand cultures was determined using the flow cytometry methods outlined in Neiman et al. (2011); only triploid (asexual) lineages were used in this experiment. All invasive populations of *P*. *antipodarum* are asexual triploids, with only one report of presumably tetraploid (asexual; Neiman et al., 2011) invasive individuals in the literature (Liu et al., 2012), allowing us to reasonably assume triploid status and asexual reproductive mode of invasive populations without flow cytometry. These invasive cultures are invariably all or nearly all female, in support of this assumption. All laboratory cultures were housed under standard laboratory conditions for *P*. *antipodarum*: 16°C temperature, 16 h light/8 h dark, and fed ad libitum *Spirulina* and powdered chalk 3 times per week before the start of the experiment. The cultures were housed in these conditions for at least 3 years prior to our experiment, providing ample time to control for any lingering maternal effects of source population on snail phenotype.

### 2.2 Lineage selection and experimental setup/procedures

We selected three asexual native lineages and three asexual invasive lineages of *P*. *antipodarum* for our experiment (mentioned above). We chose lineages (1) with sufficient numbers of juvenile snails for the experiment and (2) that prior phylogeographic (Städler et al., 2005) and (3) population genetic (Donne et al., 2020; Paczesniak et al., 2013) data suggested would be genetically distinct. We then haphazardly selected 54 juvenile snails from each lineage, establishing three replicates of “low” density (2 snails per cup; 1 snail per 150,000 mm^3^ water), “intermediate” density (6 snails per cup; 1 snail per 50,000 mm^3^ water), and “high” density (10 snails per cup; 1 snail per 30,000 mm^3^ water) treatments per lineage, for a total of 324 snails. We established our density treatments based on Zachar and Neiman (2013), who demonstrated significantly higher individual growth rates under low density (2–3 snails) relative to high population density (10–15 snails) and showed that the highest reproductive output occurred under intermediate density (4–8 snails). To track individuals, we marked each snail within a cup with a unique color of nail polish.

Each replicate of each lineage/density combination was housed in a 16‐ounce polystyrene deli cup (total volume of 641,339.7 mm^3^) filled with 300 mL of carbon‐filtered water. The water in each cup was changed weekly, and we provided each snail 0.023 mg of dried *Spirulina* dissolved in water three times a week (following Zachar and Neiman, 2013). We also supplied each cup with a pinch of powdered chalk weekly to maintain shell growth. We tracked individual growth rate by using a dissecting microscope to measure the length of the shell from the tip of the apex to the edge of the aperture on a weekly basis, following multiple other growth rate studies of *P*. *antipodarum* (e.g., Larson et al., 2020; Subba et al., 2021). In addition to measuring shell length, we performed mortality checks once a week; snails that died during the experiment were replaced by a similarly sized member of the same lineage in order to maintain experiment population density. Data from snails that died at any time during the experiment were removed from subsequent analyses. We checked each cup for newly produced offspring on a biweekly basis throughout the experiment to estimate when the majority of snails had reached sexual maturity. The experiment continued for 11 months (September 2019–August 2020), when all but 11 snails had reached 3 mm in shell length, the commonly applied threshold for sexual maturity in female *P*. *antipodarum* (e.g., Larkin et al., 2016). We calculated individual growth rate as $\left(3\;\mathrm{mm}-\text{initial shell length}\right)/\left(\text{days until shell length reached}\;3\;\mathrm{mm}\right)$ and used this measure of growth rate as a dependent variable for subsequent analyses. We measured the shell length of the snails for a final time immediately preceding dissection for embryo counts, which took place within a one‐week time span in August 2020. We dissected out the brood sac from reproductively active females using forceps under a dissecting microscope. Embryos were removed from the brood sac with forceps and counted, following approaches used in Neiman (2006) and McKenzie et al. (2013). We used embryo number (“embryo production”) and embryo presence versus absence (“reproductive status”) as dependent variables in subsequent analyses as measures of reproductive output.

### 2.3 Statistical analysis

#### 2.3.1 Growth rate

A total of 71 snails died during the experiment and were removed from growth rate analyses (see analyses of factors potentially connected to mortality in Appendix S1). Because none of the snails from the invasive lineage US1 survived in the low‐density treatment, we removed all 54 US‐1 snails from the growth‐rate analysis. We also excluded data from 12 snails that had not reached 3 mm in shell length by the end of the experiment and from 11 snails that we belatedly realized started the experiment at or above 3 mm in shell length, leaving us with a sample size of 193 snails for growth rate analyses (see Growth dataset in Table S3).

We assessed the effects of invasion status and density on growth rate by using a generalized linear mixed‐model (GLMM) with gamma distribution and log‐link function. Because Kolmogorov–Smirnov (KS) and Shapiro–Wilks (SW) analyses revealed that the individual growth rate data were not normally distributed (*p* < .001 in both cases), we applied this GLMM, which can accommodate dependent variables that that do not meet normality assumptions. This model included the fixed factors of population source (“invasion status”; invasive vs. native) and population density (“density”; low vs. intermediate vs. high) and the interaction term of invasion status by density. We included lineage nested within invasion status as a random factor. Finally, we also included initial length as a covariate in the model to account for the likelihood that variation in length could be associated with individual growth rate (e.g., Larkin et al., 2016). We used IBM SPSS 29.0 (IBM, 2023) on a Windows operating system for all analyses.

#### 2.3.2 Embryo production

After excluding 217 snails that did not produce embryos during the experiment, along with 2 snails that finished the experiment smaller than 3 mm (i.e., might not have achieved reproductive maturity) and 1 snail that started the experiment at 3 mm (large enough that growth rate data might not be reliable), we were left with a sample size of 81 reproductively active snails for analysis of embryo production. The 71 snails that died at any time during the experiment were excluded from this dataset under the presumption that tissue decomposition might prevent accurate embryo counts or definitive determination of reproductive status (see Reproduction (embryo count) dataset in Table S3). Embryo production was not normally distributed (KS: *p* < .001; SW: *p* < .001), so we assessed the effect of invasive versus native status and density on embryo production in a generalized linear mixed‐model assuming a negative binomial distribution (log link), which allows us to accommodate dependent variables that that do not meet normality assumptions. The GLMM had the fixed factors of invasion status (invasive vs. native) and density (high vs. intermediate vs. low), with the random factor of lineage (nested within invasion status), final length as a fixed covariate, and the interaction term of invasion status by density. In light of the frequently observed positive relationship between shell length and fecundity in *P*. *antipodarum* (e.g., McKenzie et al., 2013), we included final length as a covariate to account for this potentially confounding effect.

#### 2.3.3 Reproductive status

To further investigate potential density effects on reproduction, we assessed whether density treatments influenced whether adult snails produced embryos. We removed 12 snails that had a final length smaller than 3 mm, which is the minimum length threshold assumed for sexual maturity, as well as the 71 snails that died during the experiment, leaving us with 242 snails for analysis (see Reproduction (presence vs. absence) dataset in Table S3). We assessed how invasive versus native status and density affected the frequency of reproducing versus non‐reproducing snails in a generalized linear mixed‐model assuming a binary distribution (logit link) and with the fixed factors of invasion status (invasive vs. native) and density (high vs. intermediate vs. low), with the random factor of lineage (nested within invasion status), the interaction term of invasion status by density, and final length as a covariate.

## 3 RESULTS

### 3.1 Growth rate

**FIGURE 1:**
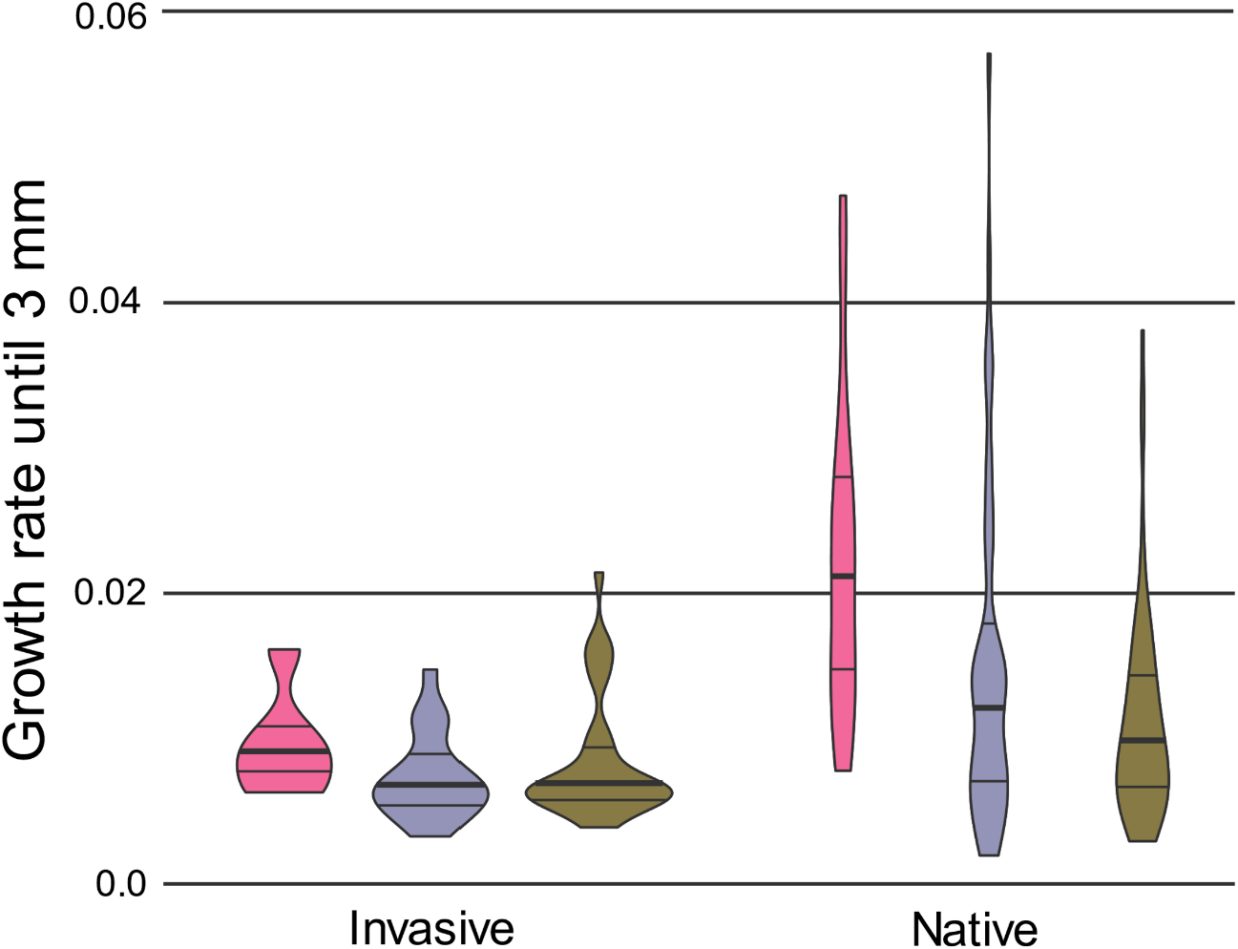
Violin plots of individual growth rate until 3 mm under low (pink), intermediate (purple), and high (green) density treatments for invasive versus native lineages; the bolded line within each violin plot represents median growth rate.

There was a significant effect of population density (*p* = .014) and a significant interaction effect of invasive status by density (*p* = .013) on individual growth rate (Table 1). The growth rate of snails in low density (median = 0.0143 mm/day; standard deviation (SD) = 0.0112) was 1.6× higher than for snails in the intermediate‐density treatment (median = 0.0089 mm/day; SD = 0.0106) and 1.74x higher than snails in the high‐density (median = 0.0082 mm/day; SD = 0.0062) treatment. We did not detect significant effects of lineage (*p* = .279), initial length (*p* = .940), or native versus invasive status (*p* = .058) on individual growth rate. The relatively low *p‐*value recovered for the native versus invasive status analysis did highlight the lower and less variable growth rate of invasive snails (median = 0.0065 mm/day; SD = 0.0038) relative to native snails (median = 0.0107 mm/day; SD = 0.0099). A post‐hoc comparison of the 95% confidence interval around these medians, as implemented in Microsoft Excel v.2312 (2023), indicated that they did not overlap (invasive = 0.0075 ± 0.00098; native = 0.0108 ± 0.00169), suggesting that the invasive snails did indeed grow at a lower rate. We interpret this outcome to indicate that the interaction effect appeared to be driven by greater sensitivity to changes in density in native versus invasive snails (Figure 1 and Figure S2).

**TABLE 1:** Results of a generalized linear mixed‐model (GLMM) with gamma distribution and log‐link function evaluating factor effects on individual growth rate.

Factors	$F_{\mathrm{df}}$ or *Z*	*p*
Invasion Status	$4.160_{\left(1,186\right)}$	.058
Density	$4.384_{\left(2,\;186\right)}$	.014
Invasion Status × Density	$4.608_{\left(2,\;186\right)}$	.013
Initial length	$0.162_{\left(1,\;186\right)}$	.940
Lineage (Invasion Status)	1.082	.279

*Note*: Invasion status and density are fixed factors. Initial shell length is a covariate. Lineage was modeled as a random factor nested in invasion status.

We further evaluated this pattern by using gamma‐distributed generalized linear models (GLM) with growth rate as our dependent variable and with density as a fixed factor performed separately in native and invasive snails. The GLM revealed a significant effect of density on the growth rate of native snails (*p* < .001) but not invasive snails (*p* = .327; Table 2). We next performed least significant difference (LSD) post‐hoc pairwise analyses of estimated marginal means to assess which density treatments performed differently. The pairwise analyses indicated significant differences in growth rate between all levels of the density treatment (high vs. intermediate: *p* = .014; intermediate vs. low: *p* = .036; high vs. low: *p* < .001) within native lineages. The growth rate of native snails in low density (median = 0.0214 mm/day; SD = 0.0111) was nearly twofold higher than the growth rate of native snails in intermediate density (median = 0.0131 mm/day; SD = 0.0118) and almost 2.5‐fold higher than native snails in the high‐density treatment (median = 0.0088 mm/day; SD = 0.0068; Table 3). By contrast, the growth rate of invasive lineages did not differ between any of the density contrasts (high vs. intermediate: *p* = .256; intermediate vs. low: *p* = .211; high vs. low: *p* = .505). The growth rate of invasive snails in low density (median = 0.0088 mm/day; SD = 0.0036) was higher than the growth of invasive snails in the intermediate (median = 0.0065 mm/day SD = 0.0030) and high‐density treatments (median = 0.0066 mm/day; SD = 0.0042).

**TABLE 2:** Results of a gamma‐distributed generalized linear model (GLM) evaluating density treatment on individual growth rate; split by invasion status (invasive vs. native).

Invasion Status	Source	Wald Chi‐square	df	*p*
Invasive	Density	2.233	2	.327
Native	Density	20.415	2	<.001

**TABLE 3:** Outcome of LSD pairwise‐post hoc comparisons of growth rate between invasive versus native lineages following the GLM described in results.

Invasion Status	Density comparisons	Mean difference	Standard error	df	*p*
Invasive	High vs. Low	−0.0011	0.0017	1	.505
	High vs. Intermediate	0.0010	0.0009	1	.256
	Low vs. Intermediate	0.0021	0.0017	1	.211
Native	High vs. Low	−0.0112	0.0035	1	<.001
	High vs. Intermediate	−0.0038	0.0015	1	.014
	Low vs. Intermediate	0.0074	0.0035	1	.036

### 3.2 Embryo production

**FIGURE 2:**
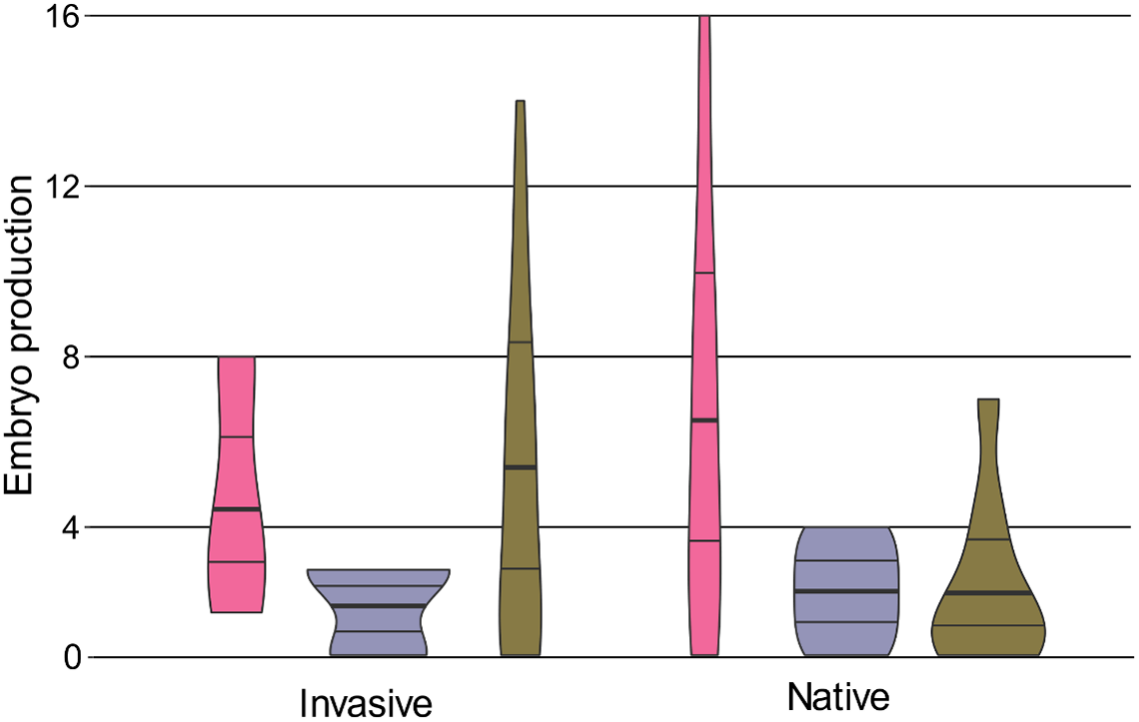
Violin plots of embryo production (number of embryos produced) per female under low (pink), intermediate (purple), and high (green) density; the bolded line within each violin plot represents median embryo production.

The GLMM revealed that neither density (*p* = .516), native versus invasive status (*p* = .732), final length (*p* = .099), nor lineage (*p* = .257) affected embryo production (Table 4). While invasive status alone did not significantly affect embryo production, invasive snails (total of 194 embryos) produced more embryos than native snails (total of 150 embryos). By contrast, embryo production was significantly affected by the interaction between invasion status and density (*p* = .033). We further investigated this interaction (Figure 2 and Figure S3) by separating the native and invasive datasets and then performing negative binomial‐distributed GLMs with embryo production as the dependent variable and density as a fixed factor for each dataset. These analyses did not detect a significant density effect on invasive snails (*p* = .336) but did reveal a marginally significant effect of density treatment on embryo production for native snails (*p* = .052; Table 5). The post‐hoc LSD pairwise comparisons of density treatments separated by invasive versus native status show no significant differences (Table S2). Across native lineages, median embryo production was highest at low density (median = 5 embryos/snail; SD = 4.9818), while embryo production at intermediate (median = 2.5 embryos/snail; SD = 1.1952) and high density (median = 2 embryos/snail; SD = 1.9029) was relatively low. By contrast, for invasive lineages, embryo production was highest at high density (median = 5 embryos/snail; SD = 3.9271), while embryo production at intermediate (median = 3 embryos/snail; SD = 1.0954) and low density (median = 4 embryos/snail; SD = 3.0551) was relatively low.

**TABLE 4:** Results of a generalized linear mixed‐model (GLMM) with a negative binomial distribution and log‐link function evaluating factor effects on embryo production.

Source	$F_{\text{df}}\;\text{or}\;Z$	*p*
Invasion Status	$0.118_{1,74}$	.732
Density	$0.668_{2,74}$	.516
Invasion Status × Density	$3.559_{2,74}$	.033
Final length	$2.796_{1,74}$	.099
Lineage (Invasion Status)	1.134	.257

*Note*: Invasion status and density are fixed factors. Final shell length is a covariate. Lineage was modeled as a random factor nested in invasion status.

**TABLE 5:** Results of a negative binomial‐distributed generalized linear model (GLM) evaluating the fixed factor of density treatment on embryo production; split by invasion status (invasive vs. native).

Invasion Status	Source	Wald Chi‐square	df	*p*
Invasive	Density	2.182	2	.336
Native	Density	5.907	2	.052

### 3.3 Reproductive status

Approximately 66% of the snails (160/242) did not produce embryos (data from the Reproduction (embryo count) dataset from Table S3). Population density affected reproductive status (*p* = .007), and there was a significant interaction between density and reproductive status (*p* = .026). Snails were more likely to be reproductive in low density (66.67%) compared to high (38.97%) and intermediate density (15.9%), though interpretation of this result is somewhat complicated by the much higher number of snails in the experiment in high and intermediate versus low‐density treatments (Table S3). The covariate of final length (*p* = .008) also had a significant influence on embryo presence versus absence. Reproductive snails (median = 4 mm; SD = 0.2863) achieved a larger size at the end of the experiment than non‐reproductive snails (median = 3.75 mm; SD = 0.3819). Neither native versus invasive status (*p* = .966) nor lineage (*p* = .248) significantly influenced reproductive status (Table 6), though a higher percentage of invasive snails were reproductive (41.1%) than native snails (28.7%). We proceeded to further investigate the density and invasion status effects by splitting the native and invasive datasets and then performing binary log‐link GLMs on each dataset with reproductive status as the dependent variable and population density as a fixed factor. Because final length was a significant covariate in the previous analysis, we included final length as a covariate in this GLM. We found that there was a significant influence of density (*p* = .002) but not final length (*p* = .650) on reproductive status for invasive snails (Table 7). Both density (*p* = .022) and final length (*p* = .030) affected reproductive status in native snails.

**TABLE 6:** Results of a generalized linear mixed‐model (GLMM) with a binary distribution and log‐link function evaluating factor effects on reproductive status.

Source	$F_{\text{df}}\;\text{or}\;Z$	*p*
Invasion Status	$0.002_{1,\;235}$	.966
Density	$5.051_{2,\;235}$	.007
Invasion Status × Density	$3.722_{2,\;235}$	.026
Final length	$7.207_{1,\;235}$	.008
Lineage (Invasion Status)	1.156	.248

*Note*: Status and density are fixed factors. Final length is a covariate. Lineage was modeled as a random factor nested in status.

**TABLE 7:** Results of a negative binary logistic‐distributed generalized linear model (GLM) evaluating the fixed factor of density treatment on reproductive status; split by invasion status (invasive vs. native).

Invasion Status	Source	Wald Chi‐square	df	*p*
Invasive	Density	12.084	2	.002
Invasive	Final length	0.206	1	.650
Native	Density	7.637	2	.022
Native	Final length	4.703	1	.030

**FIGURE 3:**
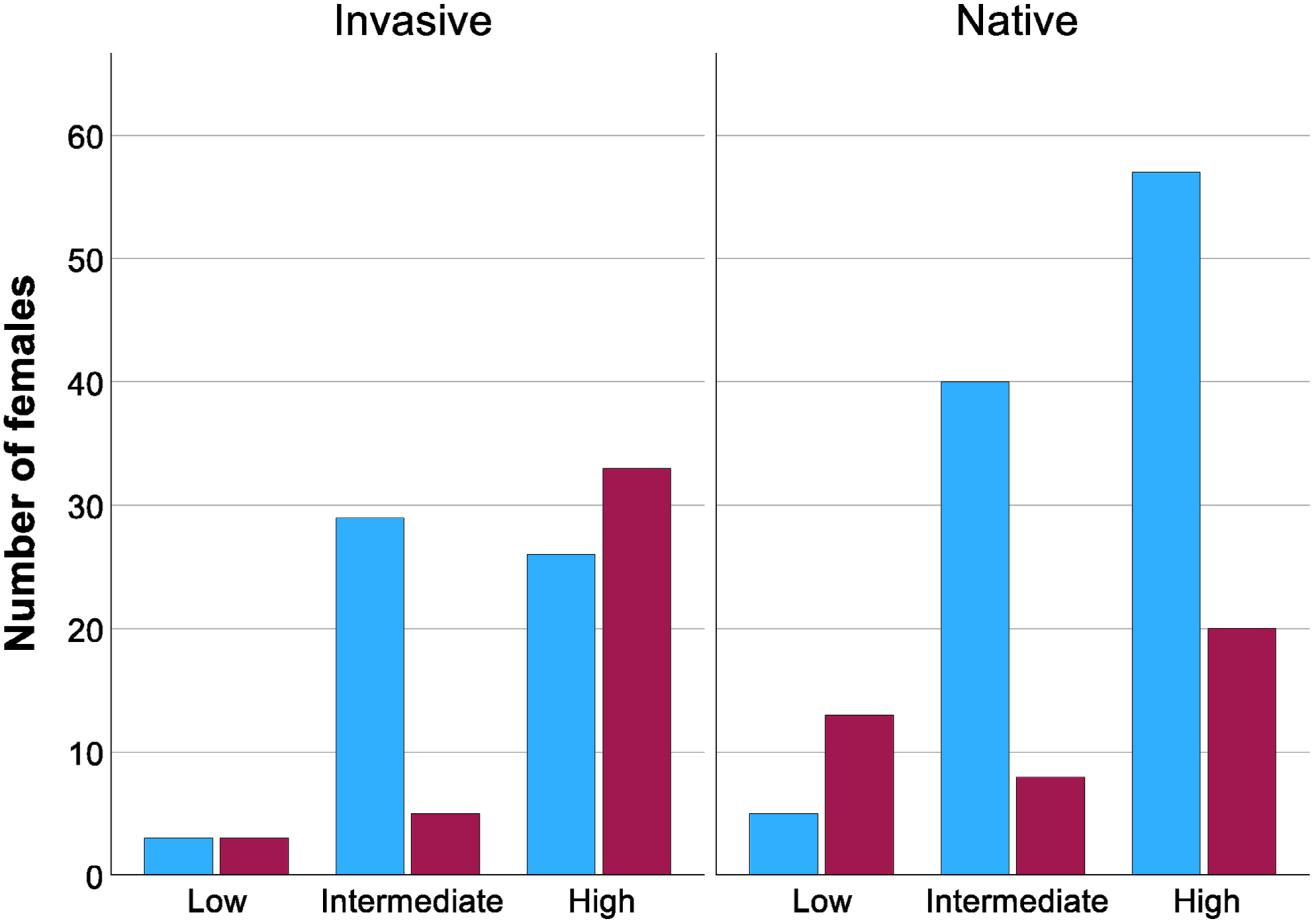
Reproductive status across density treatments and invasive versus native snails. Snails with embryos present upon dissection are represented in red. Snails without embryos present at dissection are represented in blue.

LSD post‐hoc pairwise comparisons revealed significant differences in reproductive status between high versus intermediate density treatments (*p* < .001) in invasive snails, but not in the high versus low (*p* = .727) or low versus intermediate density treatments (*p* = .135; Table 8; Figure 3). There were considerably more invasive non‐reproductive snails in intermediate density (85.3%) compared to the numbers of non‐reproductive snails in the low‐ (50%) and high‐density treatment (44.1%). For the native snails, there were significant pairwise contrasts across high versus low density (*p* = .015) and low versus intermediate density (*p* = .007), but not the high versus intermediate‐density treatment (*p* = .422). These results were driven by the much larger proportion of native snails that did not reproduce in the high density (74%) or intermediate‐density treatments (83.3%) relative to low density (27.8%).

**TABLE 8:** Outcome of pairwise post‐hoc comparisons (LSD) for reproductive status following the GLM that was separated by native versus invasive status, with the fixed factors of density and final length.

Invasion Status	Density	Mean difference	Standard error	df	*p*
Invasive	High vs. Low	−0.08	0.217	1	.727
	High vs. Intermediate	−0.40	0.091	1	<.001
	Low vs. Intermediate	−0.33	0.220	1	.135
Native	High vs. Low	0.35	0.145	1	.015
	High vs. Intermediate	−0.06	0.078	1	.422
	Low vs. Intermediate	−0.44	0.153	1	.007

## 4 DISCUSSION

We used experimental manipulation of native versus invasive lineages of *Potamopyrgus antipodarum*, a destructive worldwide invader (Nentwig et al., 2018), to address a potential role for differential native versus invasive life‐history response to population density in driving successful invasion of lineages. As predicted under a framework where relative insensitivity to density might help facilitate invasion, we found (1) that the growth of invasive snails was less sensitive to population density than their native counterparts, (2) relatively high embryo production by invasive lineages in high density, (3) a higher percentage of reproductive snails in high versus intermediate‐density treatments across invasive lineages, and (4) a higher percentage of non‐reproductive snails in the intermediate and high‐density treatments versus low‐density conditions across native lineages. These results have the potential to aid efforts to control invasive populations of *P*. *antipodarum* by informing eradication or control tactics based on life‐history trait responses to population density by following the framework discussed in Green and Grosholz (2021) and other similar works. These outcomes also suggest value in addressing the role of density dependence in population dynamics of other invasive freshwater taxa.

The mechanism(s) that influence this differential sensitivity to density‐dependent effects in *P*. *antipodarum* are not yet well characterized. One potential explanation is pheromone‐mediated communication, which is likely how *P*. *antipodarum* and other molluscan species communicate. In particular, Neiman et al. (2013) showed that the positive effects of high density on reproduction in *P*. *antipodarum* can be recapitulated in low‐density populations by transfer of water from high‐density populations, suggesting potential mediation by water‐borne and perhaps mucus‐secreted chemicals. There are multiple lines of evidence suggesting that at least some behavioral traits displayed in both freshwater and terrestrial gastropods seem to be directed by substances produced by sympatric conspecifics. For example, *Biomphalaria glabrata*, an intermediate host of *Schistosoma mansoni*, is known to gravitate toward the endogenously produced chemoattractant *Bg*Temptin, versus otherwise attractive food options when placed in a T‐maze assay (Pila 2017). Bergey et al. (2023) showed that individual *Cornu asperum* preferred mucus of *C*. *asperum* compared to mucus produced by other terrestrial snail species. In addition, *Rumina decollata*, a predatory snail species, orients preferentially to mucus produced by prey snail species rather than to conspecific‐produced mucus, possibly as a strategy to track prey. Finally, *Physa gyrina*, a freshwater snail, can use water‐borne and mucus‐produced pheromones to discriminate between well‐fed or starved conspecifics (Larcher & Crane, 2015). Altogether, these studies along with the data presented here indicate that the mechanisms that drive density‐dependent effects should continue to be a focus for research in mollusks, and perhaps especially for invasive taxa.

It is difficult to exclude the possibility that food limitation in the setting of high density could have contributed to experiment outcomes, even though we did supply each replicate with the same amount of food per snail. In particular, if variance in consumption increased with density, we might expect that some snails would suffer from food limitation at higher densities. Indeed, *P*. *antipodarum* foraging behavior is density dependent, with snails spending more time foraging at high densities (Hansen et al., 2016). There is evidence from other mollusk species of increased intensity of exploitative food competition at higher population densities. For example, *Batillaria attramentaria*, an invasive snail, outcompetes *Cerithidea californica*, a native counterpart, through food exploitation (Byers, 2000). From the same perspective, if the snails we used to replace snails that died during the experiment ate more than their deceased predecessor, perhaps as expected if snails that died were sick or otherwise did not behave normally prior to death, these replacement snails could have negatively affected growth and reproduction of their cup‐mates.

### 4.1 Growth rate

The individual growth rate of native *P*. *antipodarum* lineages decreased with increasing population density. We did not observe negative density‐dependent effects on growth rate in their invasive counterparts. These contrasting outcomes suggest that growth of invasive lineages may be relatively resilient to consequences of increasing population density. Larkin et al. (2016) found that more rapidly growing *P*. *antipodarum* achieved reproductive maturity at an earlier age, hinting that the ability to maintain growth at high density could translate into relatively early age at first reproduction, with the potential for higher lifetime reproductive fitness (MacArthur & Wilson, 1967). Because growth rate and size at reproduction are positively correlated in adult female *P*. *antipodarum* (Donne et al., 2022), and relatively large *P*. *antipodarum* produce relatively more embryos (McKenzie et al., 2013; Tibbets et al., 2010), the growth‐rate resilience that we observed in invasive lineages could indeed have multiple positive downstream consequences for fitness. This result supports the possibility that the successful invasion of certain *P*. *antipodarum* lineages may, in part, be linked to tolerance to potentially stressful environmental factors, such as relatively high or low population density. Consistent with this hypothesis, invasive *P*. *antipodarum* are more tolerant to phosphorus limitation than their native counterparts (Neiman & Krist, 2016).

In particular, we have shown that invasive and native conspecifics might display differential growth responses to population density. These observations are especially intriguing in light of other evidence that invasive *P*. *antipodarum* have distinct life‐history trait values (e.g., age and size at sexual maturity and growth rate; Donne et al., 2022). One potential implication of these results is that invasive *P*. *antipodarum* may be more likely to persist through the introduction and establishment stages of invasion via growth rate insensitivity to population density. Indeed, life‐history trait plasticity and intraspecific trait variation are important determinants of a successful invasion (Davidson et al., 2011; Westerband et al., 2021). Empirical assessment of this possibility for *P*. *antipodarum* could include studies tracking population dynamics of experimental mesocosms of native versus invasive lineages as well as by direct study and comparison of the population density and dynamics of *P*. *antipodarum* in New Zealand and in invaded regions.

### 4.2 Embryo production

There was a trend toward highest embryo production for invasive *P*. *antipodarum* at high density. Our results mimic the results reported in Neiman et al. (2013), where relatively high reproductive output was found in *P*. *antipodarum* in high versus low population density. Positive density‐dependent effects on reproduction have also been reported in other invertebrate taxa (for example, the freshwater snail *Margarya melanoides* (Song et al., 2017) and the roundworm *Caenorhabditis elegans* (Wong et al., 2020)). Zachar and Neiman (2013) found that reproduction is maximized at intermediate density in two *P*. *antipodarum* lineages, suggesting that positive effects of higher density do not universally apply. If the positive density‐dependent effects, we find here are recapitulated in at least some newly invasive populations of *P*. *antipodarum*, they could decrease the likelihood that the population crashes as density increases, increasing the potential for successful establishment.

By contrast, there was a trend toward native lineages producing the most embryos in low density. This result is similar to that of Dinges and Lively (2022), where mesocosm experiments showed that reproduction of native *P*. *antipodarum* lineages was negatively affected by high population density. In our experiment, native‐lineage snails produced relatively few embryos in intermediate and high density, which echoes trends reported for native‐range *P*. *antipodarum* in Cope and Winterbourn (2004). While further work is needed to understand the mechanisms underlying these negative consequences of high density for native lineages and why they differ between invasive and native lineages, we speculate that they could include tradeoffs between life‐history trait expression and how these traits are influenced by factors that could be enhanced at high density (e.g., interspecific competition (Watz & Nyqvist, 2022); parasite transmission (reviewed in Lopez & Duffy, 2021); chemical interference (Neiman et al., 2013)).

### 4.3 Reproductive status

There was a significant difference in reproductive status across high versus low‐density treatments and intermediate versus low‐density treatments for native lineages, with more non‐reproductive females than reproductive females in intermediate and high density, and more non‐reproductive females in intermediate and high density compared to low density. By contrast, there were more reproductive females than non‐reproductive females in the low‐density treatment for native lineages. These outcomes for reproductive status mirror our embryo production results – native *P*. *antipodarum* perform better in low‐density treatments compared to intermediate and high‐density treatments (also see Cope & Winterbourn, 2004; Dinges & Lively, 2022).

The effect of population‐density treatments on reproductive status differed for invasive snails. Here, we observed an increased likelihood of embryo production in high density relative to the intermediate‐density treatment. In other words, invasive lineages do not seem to experience negative effects of high population density with respect to reproductive status. This outcome is consistent with reports that invasive *P*. *antipodarum* are less sensitive to environmental influences than native counterparts (reviewed in Geist et al., 2022). Our results did depart somewhat from a previous smaller study of density effects in an invasive lineage of *P*. *antipodarum* that found that reproduction was maximized at intermediate density (Zachar & Neiman, 2013). This difference in outcomes is perhaps unsurprising in light of the wide variation of life‐history trait expression across native and invasive *P*. *antipodarum* (Donne et al., 2022; Larkin et al., 2016; Verhaegen et al., 2021) and demonstrates the importance of surveying a diverse set of lineages when assessing density effects.

## 5 CONCLUSIONS AND FUTURE STEPS

There are multiple examples of invasive species outcompeting native species in a novel environment, such as Kentucky blue grass (*Poa pratensis* subsp. *Angustifolia*) in the prairie grasslands of Alberta, Canada (Zapisocki et al., 2022) and invasive crayfish (*Faxonius limosus*) in the Lower Danube River of Southeast Europe (Pacioglu et al., 2020). Several recent studies have demonstrated this phenomenon in invasive freshwater snails, for example, the acute bladder snail (*Physa acuta*) on the Grande‐Terre island of Guadeloupe (Dubart et al., 2019) and our focal species, the New Zealand mudsnail (*P*. *antipodarum*), in Polecat Creek in Grand Teton National Park, Wyoming (Riley & Dybdahl, 2015). It is thus perhaps not surprising that population density effects have been relatively well studied at the interspecific scale, including growth rate as a function of population density in invasive *Taraxacum officinale* versus native *Taraxacum platycarpum* (Lee et al., 2021) and in invasive *Physa acuta* versus native *Physa fontinalis* (Früh et al., 2017). Our study, which is instead focused on intraspecific density‐dependent effects, is relevant to understanding why some lineages become successful invaders while others do not, providing important insights into the evolution and ecology of biological invasions. In particular, we have documented life‐history trait differences between invasive and native lineages in the same population‐density conditions. Our results (1) demonstrate intraspecific variation in sensitivity to population density, (2) show that invasive lineages tend to be less sensitive to density than native counterparts, and (3) hint that lineage‐level variation in sensitivity to population density could help drive successful biological invasions for some *P*. *antipodarum* lineages, with the caveat that laboratory results are difficult to generalize to natural conditions.

Taken together, these findings help illuminate the invasion biology of *P*. *antipodarum* and are exciting from the perspective of providing some of the first evidence for differential sensitivity to population density in native versus invasive conspecifics. These results highlight the importance of evaluating life‐history trait variation on an intraspecific level when studying the evolution and ecology of invasion and may be directly transferrable to the many other aquatic snails that are or could be successful invaders (Preston et al., 2022). Important next steps include assessing whether other life‐history traits (e.g., time to sexual maturity; Larkin et al., 2016) are also differentially sensitive to population density across native and invasive lineages and determining whether the positive consequences of exposure to “high density” water observed in Neiman et al. (2013) are confined to invasive lineages. Repeating the experiment of Zachar and Neiman (2013), which assessed growth and reproduction of just two *P*. *antipodarum* lineages in a broad range of density treatments, across a much more diverse array of *P*. *antipodarum* would also be illuminating.

## AUTHOR CONTRIBUTIONS

**Briante Shevon Lewis Najev:** Conceptualization (supporting); formal analysis (lead); funding acquisition (supporting); methodology (equal); supervision (equal); visualization (lead); writing – original draft (lead); writing – review and editing (equal). **Maurine Neiman:** Conceptualization (lead); formal analysis (supporting); funding acquisition (lead); investigation (equal); methodology (equal); resources (lead); supervision (equal); visualization (supporting); writing – original draft (supporting); writing – review and editing (equal).

## FUNDING INFORMATION

This work was supported by Graduate College at the University of Iowa, the Office of Undergraduate Research, the Roy J. Carver Charitable Trust grant [18‐5081], and the National Center for Science Education.

## CONFLICT OF INTEREST STATEMENT

The authors have no relevant financial or non‐financial interests to disclose.

## Supporting information


Appendix S1.–S3.

Data S1.

## Data Availability

The datasets generated during and/or analyzed during the current study are available on Dryad. These data are also available in a supplemental file of this publication.
