# Supplementary material for: Invasive freshwater snails are less sensitive to population density than native conspecifics
Source: Ecol Evol. 2024 May 20;14(5):e11161. doi: 10.1002/ece3.11161 (PMC11106046; doi:10.1002/ece3.11161)
Supplement: Supplementary file 1 — Appendix S1.–S3. [file ECE3-14-e11161-s002.docx]

*Appendix S1*

**Mortality**

21.9% of the snails (71/324) died before the experiment end (dissection). Because more than 1/5 snails died throughout this experiment, we felt it was relevant to additionally evaluate mortality *a posteriori.* Snails that died or were found dead on a dissection day were considered dead. We removed snails that did not reach 3 mm by the end of the experiment (12 snails) and snails that started the experiment at or exceeding 3 mm (11 snails) from the dataset, leaving us with 301 snails. We assessed effects of invasive vs. native status and density on mortality in a generalized linear model assuming a negative binomial distribution (log link) and with the fixed factors of status (invasive vs. native) and density (high vs. intermediate vs. low), with the random factor of lineage (nested within status), and the interaction term of status vs. density.

The GLMM revealed that neither density (*p* = 0.677), native vs. invasive status (*p* = 0.958), lineage (*p* = 0.204), nor the interaction between density and status (*p* = 0.784) affected mortality (Table S1; Fig. S1). Because none of the main effects were significant, we did not investigate mortality any further. However, we would like to note a few trends: 1) more invasive snails died compared to native snails, 2) more invasive snails in low density died than survived throughout the experiment, and 3) no native snails died in the low-density treatment, with the caveat that there were many fewer total snails in this treatment relative to intermediate and high density (Fig. 2).

**Table S1** Results of a generalized linear mixed-model (GLMM) with a binomial distribution and log-link function evaluating factor effects on mortality. Status, density, and status by density are fixed factors. Lineage was modeled as a random effect nested in status.

| Source | $F_{\mathrm{df}} \mathrm{or} Z$ | *p* |
| --- | --- | --- |
| Status | ${0.003}_{1, 295}$ | 0.958 |
| Density | ${0.391}_{2, 295}$ | 0.677 |
| Status * Density | ${0.243}_{2,295}$ | 0.784 |
| Lineage (Status) | 1.271 | 0.204 |


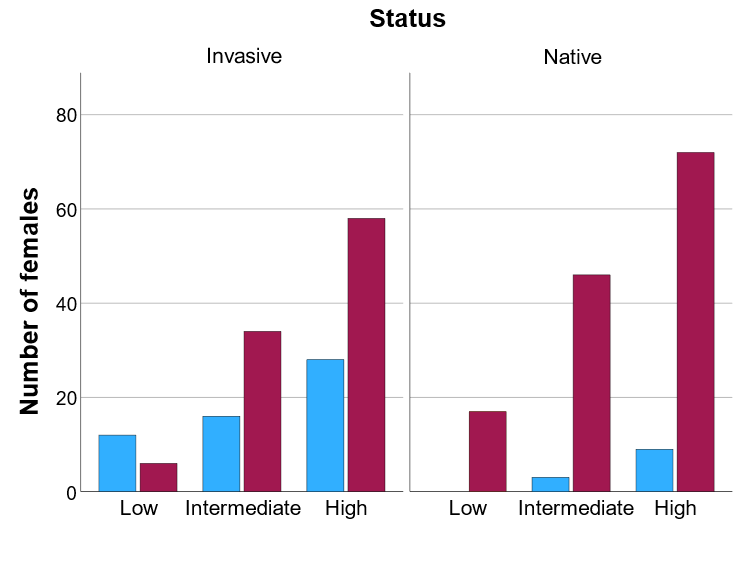


**Fig. S1** Snail mortality across density treatments and invasive vs. native status. Snails that died at any time during the experiment were considered dead, represented in blue. Snails classified as alive survived throughout the whole experiment, represented in red.

*Appendix S2*

**Table S2** Outcome of pairwise-posthoc comparisons (LSD) for embryo production following the negative binomial-distributed generalized linear model GLM of Table 5, separated by native vs. invasive status, with the fixed factors of density.

| Status | Density | | Mean Difference | Standard Error | df | *p* |
| --- | --- | --- | --- | --- | --- | --- |
| Invasive | | High v. Low | -0.45 | 3.125 | 1 | 0.884 |
|  |  | High v. Intermediate | -2.92 | 1.536 | 1 | 0.057 |
| Native | | Low v. Intermediate | -2.47 | 3.197 | 1 | 0.440 |
|  |  | High v. Low | -3.90 | 2.128 | 1 | 0.067 |
|  |  | High v. Intermediate | -0.10 | 1.250 | 1 | 0.936 |
|  | | Low v. Intermediate | -4.00 | 2.271 | 1 | 0.078 |

**Table S3** The number of snails used for data analyses for all variables and factors (e.g., 301 snails used to test effects on mortality).

| Dataset | Status | Density | Snail number |
| --- | --- | --- | --- |
| Original data set | Invasive | Low | 18 |
|  |  | Intermediate | 54 |
|  |  | High | 90 |
|  | Native | Low | 18 |
|  |  | Intermediate | 54 |
|  |  | High | 90 |
| Mortality | Invasive | Low | 18 |
|  |  | Intermediate | 50 |
|  |  | High | 86 |
|  | Native | Low | 17 |
|  |  | Intermediate | 49 |
|  |  | High | 81 |
| Growth | Invasive | Low | 6 |
|  |  | Intermediate | 19 |
|  |  | High | 33 |
|  | Native | Low | 17 |
|  |  | Intermediate | 46 |
|  |  | High | 72 |
| Reproduction (embryo count) | Invasive | Low | 3 |
|  |  | Intermediate | 5 |
|  |  | High | 33 |
|  | Native | Low | 12 |
|  |  | Intermediate | 8 |
|  |  | High | 20 |
| Reproduction (presence vs. absence) | Invasive | Low | 6 |
|  |  | Intermediate | 34 |
|  |  | High | 59 |
|  | Native | Low | 18 |
|  |  | Intermediate | 48 |
|  |  | High | 77 |

*Appendix S3*

*
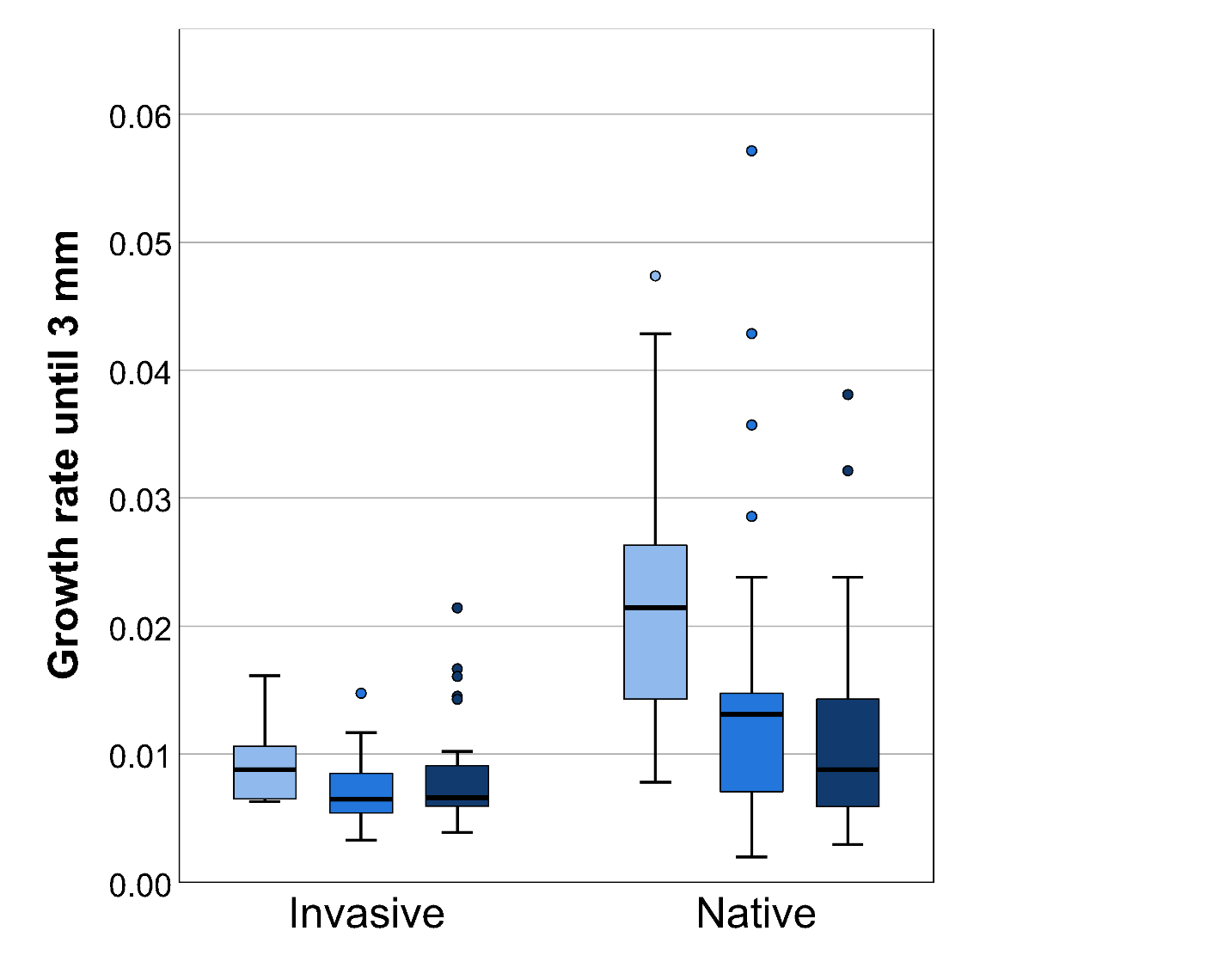
*

**Fig. S2** Box plots of individual growth rate until 3 mm under low (light blue), intermediate (medium blue), and high (dark blue) density treatments for invasive vs. native lineages; the bolded line within each box plot represents median growth rate. The points on this graph represent outliers.


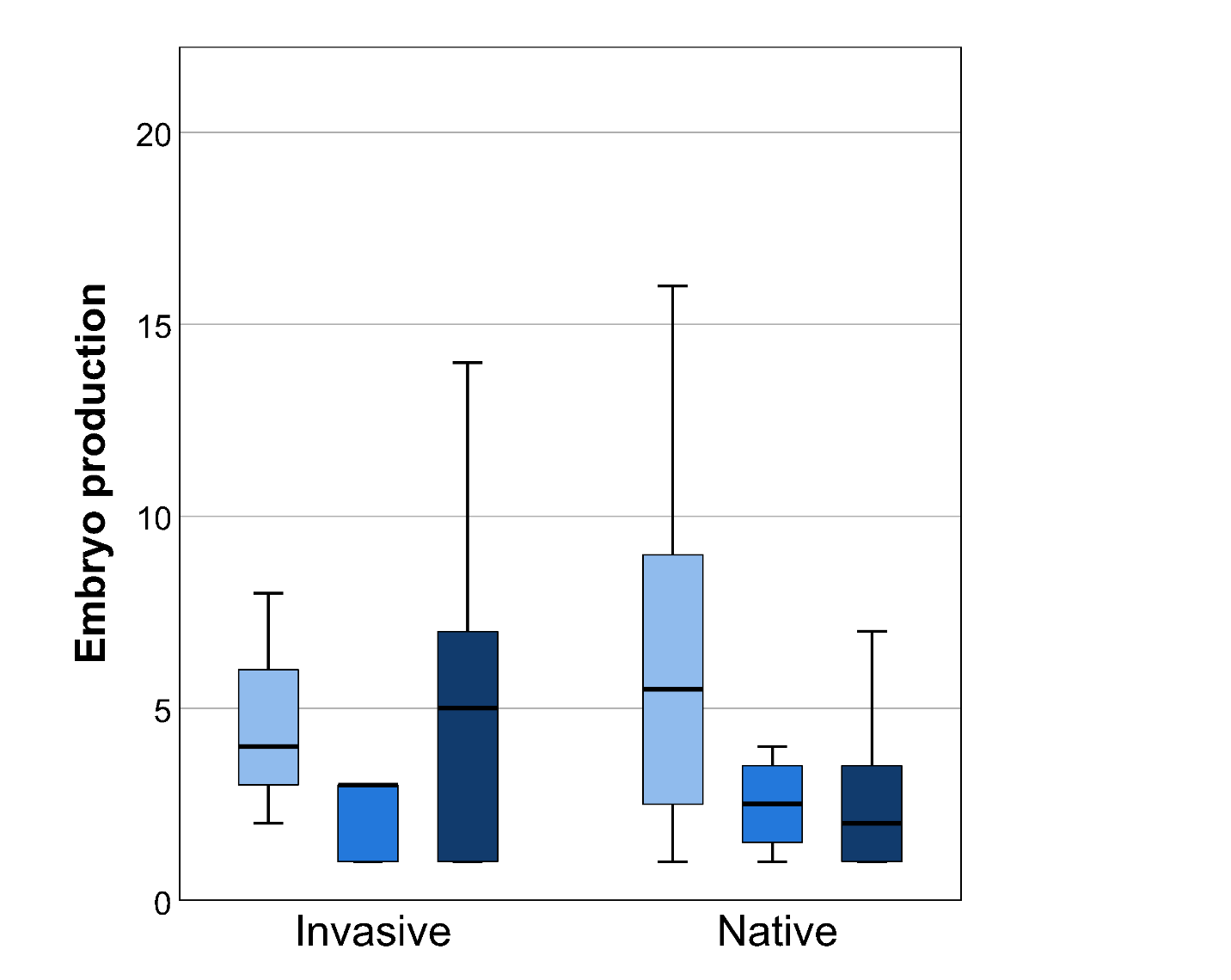


**Fig. S3** Box plots of embryo production (number of embryos produced) per female under low (light blue), intermediate (medium blue), and high (dark blue) density treatments for invasive vs. native lineages; the bolded line within each box plot represents median embryo production.
